# Acute free perforation of gall bladder encountered at initial presentation in a 51 years old man: a case report

**DOI:** 10.1186/1757-1626-2-166

**Published:** 2009-10-26

**Authors:** Abdul Rehman Alvi, Saad Ajmal, Taimur Saleem

**Affiliations:** 1 Department of Surgery, Section of General Surgery, The Aga Khan University, (Stadium Road), Karachi, (74800), Pakistan; 2 Medical College, The Aga Khan University, (Stadium Road), Karachi, (74800), Pakistan

## Abstract

**Introduction:**

Gallbladder perforation is a rare but life threatening event. We describe a case of gallbladder perforation encountered at initial presentation.

**Case presentation:**

A 51 years old male, without any known medical co-morbidity, presented with a 1-day history of sudden-onset abdominal pain and abdominal distension. On examination, his abdomen was distended with generalized tenderness on palpation. Abdominal x-ray showed no signs of intestinal obstruction or pneumoperitoneum. Computed tomography scan of the abdomen showed appearance suggestive of gallbladder perforation. The patient was taken to the operating room and a diagnostic laparoscopy was performed revealing yellowish green fluid in the peritoneum. Difficulty in visualization of the anatomy led to conversion of the procedure to an open laparotomy. Intra-operative findings included a perforation near the neck of the gall bladder in association with a 2 × 1 cm gall stone. Near-total cholecystectomy was performed and a single large gall stone was retrieved. The peritoneal cavity was washed with normal saline and a drain was placed. The rectus sheath was closed but the wound was kept open for healing by delayed primary closure. The patient's hospital course was uneventful and he was discharged from the hospital on the 3^rd ^post-operative day. He returned to the clinic after one week whereby his drain was removed and his wound closed.

**Conclusion:**

Gallbladder perforation is an unusual initial presentation of gallbladder disease. Early diagnosis of gallbladder perforation and immediate surgical intervention are of prime importance in decreasing morbidity and mortality associated with this condition.

## Introduction

Empyema, gallstone ileus, cholecystoenteric fistula, emphysematous cholecystitis, gallbladder perforation and biliary peritonitis are among the severe complications of acute calculous cholecystitis. These complications are associated with increased morbidity and mortality [1], and can develop at a high rate if the condition is left untreated.

According to one study, 12 (3.3%) cases of acute cholecystitis were complicated by gall bladder perforation out of a total of 386 patients [1], while another study has reported the incidence of gall bladder perforation complicating acute cholecystitis in 5.9% of 31 patients [2]. Gall bladder perforation has also been reported in literature with acute acalculous cholecystitis but at a lower rate [3].

We report here the case of a 51 years old gentleman who presented with acute free perforation of the gall bladder and associated biliary peritonitis in the absence of any previous clinical episodes of acute cholecystitis.

## Case presentation

A 51-years-old Pakistani male, without any known medical co-morbidity, presented to the emergency department at our institution with a 1 day history of sudden-onset and severe abdominal pain along with abdominal distension. The pain had started after a long drive of 12 - 14 hours. It was initially localized to the right upper quadrant and right lumbar region but had evolved to a more generalized distribution over time. The pain was aggravated on movement and relieved by rest. There were no other associated symptoms. His past surgical history was significant for a repaired left sided inguinal hernia 10 years back.

The patient was in obvious anxiety and distress when first encountered in the emergency room. His vital signs were stable except for an increased pulse of 105 beats per minute. Examination of his respiratory system revealed vesicular breathing with decreased intensity of breath sounds in the right lung base as well as dullness on percussion on the right side of the chest. His abdomen was distended with generalized tenderness on palpation and minimal movement with respiration. No organomegaly was appreciated on the physical examination. His bowel sounds were sluggish.

**Figure 1:**
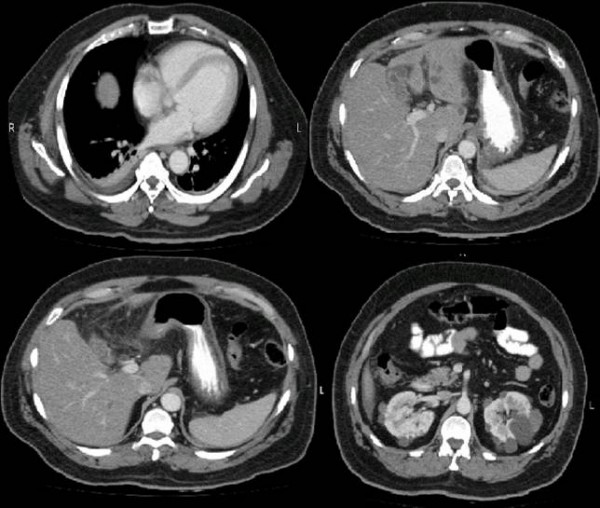
**Cross sectional view of abdominal CT scan showing indistinct margins of gallbladder, pericholecystic fat stranding and streaks of fluid**.

**Figure 2:**
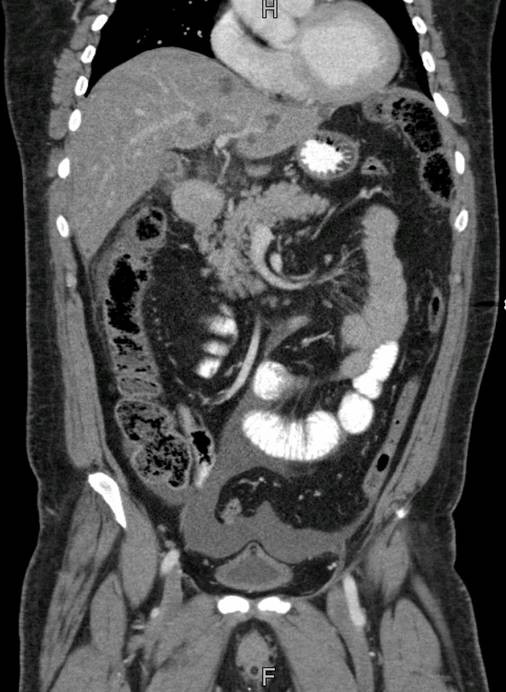
**Sagittal section of abdominal CT scan showing pericholecystic fat stranding, and mild fluid in the pelvis**.

The laboratory tests done at the time of admission are shown in table 1. The patient had mild anemia, mildly reduced hematocrit, leukocytosis with neutrophilia and hyponatremia. His serum amylase, lipase and random blood sugar levels were all within normal limits. His abdominal x-ray showed sub-optimal inspiration with minimal right sided pleural effusion. However, there were no signs of intestinal obstruction or pneumoperitoneum noted. Subsequently, computed tomography (CT) scan of the abdomen was done. Gallbladder margins were not very clearly identifiable; there was significant pericholecystic fat stranding with pericholecystic fluid. A circular high density focus in the proximity of gallbladder was identified which was thought to be representing a gallstone. Mild ascites was also present. These features were collectively suggestive f gallbladder perforation. (Figures 1 and 2)

**Table 1:** Laboratory parameters of the patient at the time of admission

Laboratory test	Result	Normal reference range (for healthy males)
Hemoglobin (g/dl)	13.1	13.7 - 16.3
Hematocrit (%)	39.4	41.9 - 48.7

White blood cell count (× 10^9^/L)	16.6	4 - 10
Neutrophils (%)/Lymphocytes (%)	91.3/7	40 - 75/20 - 45

Platelets (× 10^9^/L)	237	150 - 400

Serum creatinine (mg/dl)	1.0	0.85 - 1.35
Blood Urea Nitrogen (mg/dl)	14	6 - 16

Prothrombin time (seconds)	11.3	11
INR	1.08	1
Activated partial thromboplastin time (seconds)	26.4	30

Serum Sodium (mmol/L)	132	136 - 148
Serum Potassium (mmol/L)	3.8	3.6 - 5
Serum Chloride (mmol/L)	101	104 - 114
Serum Bicarbonate (mmol/L)	24.8	17.5 - 27.5

Troponin I (ng/ml)	< 0.5	< 0.5

Serum Lipase (u/L)	24	12 - 70
Serum Amylase (u/L)	56	25 - 125
Random Blood Glucose (mg/dl)	124	80 - 160

The patient's emergent management included nothing per oral status, regular vitals and input/output charting, insertion of nasogastric tube, administration of intravenous fluids, narcotic analgesics, proton pump inhibitors, metochlopramide, ceftriaxone, metronidazole, and ampicillin.

The patient was then taken to the operating room and a diagnostic laparoscopy was performed. It revealed free yellowish green fluid, most likely bilious, in the peritoneum. As the triangle of Calot could not be identified on laparoscopy, the procedure was converted to an open laparotomy. Intra-operative findings included presence of frank bile in the abdomen and a thick walled gall bladder. Dense adhesions of the gall bladder with surrounding structures were observed. A small perforation was visible near the neck of gall bladder in association with a large gall stone. The anatomy of the Calot's triangle was still obscure. A near-total or partial cholecystectomy including the perforated area was performed and a single large gall stone measuring around 2 × 1 cm was retrieved. The peritoneal cavity was washed with copious amounts of normal saline and a drain was placed. The rectus sheath was closed with loop polydioxanon (PDS) but the wound was kept open for healing by delayed primary closure.

Histopathology of the specimen showed full thickness gallbladder wall lined by mucosa with focal ulceration. Rokitansky aschoff sinuses were also seen. A pathological diagnosis of acute on chronic cholecystitis was made.

The patient's hospital course was uneventful. His symptoms significantly improved and he was discharged from the hospital on the third post-operative day. He returned to the clinic after one week when his drain was removed and his wound closed.

## Discussion

Gallbladder perforation is a rare but life threatening event [4]. Broadly speaking, gallbladder perforations can be traumatic, iatrogenic, or idiopathic. Conditions such as cholelithiasis, infections, malignancy, steroid therapy, diabetes mellitus and atherosclerotic heart disease are all predisposing factors for gallbladder perforation [5].

Niemeier in 1934 classified free gallbladder perforation into 3 types. Type 1 (acute) is associated with generalized biliary peritonitis, type 2 (subacute) consists of localization of fluid at the site of perforation, pericholecystic abscess and localized peritonitis while type 3 (chronic) comprises formation of internal or external fistulae [6]. Recent studies have cited higher rates of subacute or Type 2 perforations as compared to other types [7,8]. Our patient had a type 1 gallbladder perforation because of associated generalized biliary peritonitis.

In a study reporting the features of gall bladder perforation in 19 patients with acute cholecystitis, the mean age of the patients was 69 years with a female: male ratio of 3:2. It was also seen in this study that most of the patients had a history suggestive of gallbladder disease and had medical co-morbidities such as cardiac, pulmonary, renal, nutritional or metabolic diseases. The perforation occurred in most of these patients within 72 hours [9]. According to other reports, perforation of the gallbladder can occur as early as 2 days after the onset of acute cholecystitis, or after a few weeks [5,10].

Our case is unusual because our patient was younger in comparison, had no prior history suggestive of gallbladder disease and had no known medical co-morbidity. Histopathological examination of the specimen showed features of acute-on-chronic cholecystitis leading to the derivation that the prior episodes of cholecystitis in this patient were clinically silent. Per-operatively our patient's gallbladder showed dense adhesions with the surrounding structures and no signs of gangrene of the rest of the gallbladder. This further strengthens the possibility of clinically covert episodes of acute cholecystitis in the past due to the adhesions noted; however the patient denied any past history of abdominal pain. The gallbladder perforation in our case presumably occurred within the first 12-18 hours of the onset of symptoms. In addition, the perforation occurred at the neck of the gallbladder which is an uncommonly reported site for such an event. The most common sites for the perforation of the gallbladder are the fundus and the body of the gallbladder due to poorer vascular supply of these areas as compared to the neck of the gallbladder [11].

We initially performed an x-ray of the abdomen in this patient. When no signs of pneumoperitoneum or intestinal obstruction were demonstrated, a CT scan of the abdomen was performed. In a series of 17 patients with gallbladder perforation who presented with an acute abdomen, CT scan was the preliminary radiological investigation [7]. Sensitivity of CT in the detection of gallbladder perforation and biliary calculi has been reported to be 88% and 89%; these figures are higher than those reported for ultrasonographic examination [7].

Early surgical intervention is an important step in the management of gallbladder perforation. It is important to be mindful that increased morbidity and mortality is associated with this condition because of delay in diagnosis and initiation of treatment.

## Conclusion

Early diagnosis of gallbladder perforation and immediate surgical intervention are of prime importance in decreasing morbidity and mortality associated with this condition. The presence of risk factors certainly warrants an aggressively oriented investigation stratagem to rule out this serious complication. However, it is also important to consider this condition as an important differential in patients without any prior features or history of gallstone disease but whose acute presentation may be indicative of biliary pathology as was the case in this patient.

## Abbreviations

CT: computed tomography; PDS: polydioxanon.

## Competing interests

The authors declare that they have no competing interests.

## Authors' contributions

SA and TS were involved in data collection, interpretation and writing the manuscript. ARA was involved in study conception and design, drafting the manuscript and providing overall supervision in the project. All authors read and approved the final manuscript.

## Consent

Written, informed consent was obtained from the patient for the publication of this case report and accompanying images. A copy of the consent form is available for review by the Editor-in-Chief of this journal.

## References

[B1] Bedirli A Sakrak O Sözüer EM Kerek M Güler I Factors effecting the complications in the natural history of acute cholecystitis Hepatogastroenterology 2001 48 1275 1278 11677945

[B2] Menakuru SR Kaman L Behera A Singh R Katariya RN Current management of gall bladder perforations ANZ J Surg 2004 74 843 846 10.1111/j.1445-1433.2004.03186.x 15456428

[B3] Chalupa P Kaspar M Holub M Acute acalculous cholecystitis with pericholecystitis in a patient with Epstein-Barr Virus infectious mononucleosis Med Sci Monit 2009 15 CS30 33 19179974

[B4] Derici H Kara C Bozdag AD Nazli O Tansug T Akca E Diagnosis and treatment of gallbladder perforation World J Gastroentero 2006 12 7832 7836 10.3748/wjg.v12.i48.7832 PMC4087551 17203529

[B5] Strohl EL Diffenbaugh WG Baker JH Chemma MH Collective reviews: gangrene and perforation of the gallbladder Int Abstr Surg 1962 114 1 7

[B6] Niemeier OW Acute free perforation of the gall bladder Ann Surg 1934 99 922 924 17867204 10.1097/00000658-193499060-00005 PMC1390061

[B7] Morris BS Balpande PR Morani AC Chaudhary RK Maheshwari M Raut AA The CT appearances of gallbladder perforation Br J Radiol 2007 80 898 901 10.1259/bjr/28510614 17908817

[B8] Bennet GL Balthazar EJ Ultrasound and CT evaluation of emergent gallbladder pathology Radiol Clin North Am 2003 41 1203 1216 10.1016/S0033-8389(03)00097-6 14661666

[B9] Williams NF Scobie TK Perforation of the gallbladder: analysis of 19 cases Can Med Assoc J 1976 115 1223 1225 1000456 PMC1878976

[B10] Cope Z A sign of gallbladder disease BMJ 1970 3 147 148 10.1136/bmj.3.5715.147 5431086 PMC1702308

[B11] Namikawa T Kobayashi M Okabayashi T Okamoto K Akimori T Sugimoto T Hanazaki K Clinicopathological analysis of idiopathic perforation of the gallbladder Surg Today 2007 37 633 637 10.1007/s00595-006-3476-2 17643203

